# Cytotoxicity of Self-Etch Versus Etch-and-Rinse Dentin Adhesives: A Screening Study

**DOI:** 10.3390/ma13020452

**Published:** 2020-01-17

**Authors:** Luisa Fröb, Stefan Rüttermann, Georgios E. Romanos, Eva Herrmann, Susanne Gerhardt-Szép

**Affiliations:** 1 Department of Operative Dentistry, Dental School (Carolinum), Goethe University, Theodor-Stern-Kai 7, 60590 Frankfurt am Main, Germany; s2038491@stud.uni-frankfurt.de (L.F.); ruettermann@med.uni-frankfurt.de (S.R.); 2 Department of Periodontology, School of Dental Medicine, Stony Brook University, Stony Brook, New York, NY 11794, USA; georgios.romanos@stonybrook.edu; 3 Department of Oral Surgery and Implant Dentistry, Dental School (Carolinum), Goethe University, Theodor-Stern-Kai 7, 60590 Frankfurt am Main, Germany; 4 Institute of Biostatistics and Mathematical Modelling, Goethe University, Theodor-Stern-Kai 7, 60590 Frankfurt am Main, Germany; herrmann@med.uni-frankfurt.de

**Keywords:** cells, cytotoxicity, dentin adhesives, fibroblasts, in vitro, screening

## Abstract

Six dentin adhesives were tested in vitro regarding their cytotoxicity on human fibroblasts. The adhesives Hybrid Bond, One-up Bond F Plus, AdheSE, Clearfil SE Bond, Optibond Solo Plus and Syntac were eluted with culture medium as single or sequentially applied adhesive part for 24 h. 75 Petri dishes were produced per group. They were evaluated triangulated, comprising the quantitative evaluation (105 ones) to determine “viable”, “dead” and “debris” cells with the use of a cell-counter and the reactivity index was also identified based on the qualitative assessment (420 ones). One-up Bond F Plus, AdheSE and Clearfil SE Bond showed a statistical difference of viable cells to the cell control. For One-up Bond F Plus, statistically, differences compared to hybrid bond and Syntac were also found. All the adhesives except One-up Bond F Plus showed significant differences between single and sequentially applied adhesive part regarding the quantitative evaluation. The test material showed a moderate grade of cytotoxicity. As a result, a statistically significant difference of the cytotoxicity between the self-etch and etch-and-rinse adhesives cannot be demonstrated regarding the qualitative evaluation and the reactivity index, but the differences between sequentially applied and single applied components can be proved.

## 1. Introduction

A growing demand of dentists for quicker work has led to an increasing number of applications of Self-Etch adhesives. Their application is user-friendly and less technique-sensitive [1]. They do not require separate conditioning [1,2,3,4]. Self-etch adhesive can be categorized according to the mechanism of clinical application into one-step, which is also called “all-in-one”, or two-step self-etch adhesives [1,2,5]. The conventional dentin adhesives, which need a conditioning with 37% phosphoric acid, are etch-and-rinse bonding systems [3,4,5]. There is also a sub-classification according to the number of steps, which are provided in the application [6].

Dental adhesives have intraoral direct or indirect contact to gingival and through the dentinal tubules to pulp tissue [5,7]. It is frequently argued that dental materials that come into contact with intraoral tissue may have the potential to damage it [5,8,9,10,11]. For this reason, it is important to test the cytotoxicity of these materials. In general, the test of cytotoxicity of dental material follows a specific sequence of tests according to the ISO 10993-5 [12]. First, an in vitro screening study is carried out by means of qualitative evaluation, to rank the test material [12,13]. Within the framework of a screening study, the reactivity index is also determined [12].

In previous studies, the cytotoxicity of different adhesives is discussed controversially. It is difficult to compare the evaluation of each study because the investigations considered different methods and parameters [11,14,15,16,17,18,19,20,21,22,23,24,25,26,27,28,29,30,31,32,33,34,35]. This includes that, in many studies, the individual parts of the adhesives were tested for their cytotoxicity as a single substance and not in the manner actually performed clinically [11,28,29,30]. Some dentin adhesives consist of several bottles, for example, primer and bond. The test of cytotoxicity of these individual bottles is called single applied adhesive part respectively additive analysis sequentially applied adhesive parts. In addition, the evaluation was usually only quantitative [11,15,18,19,20,21,22,23,24,25,27,28,29,31,32,33,34,35]. A combination of quantitative and qualitative assessments, which is based on ISO 10993-5, is only rarely available in the case of dental adhesives, which refer exclusively to older adhesives like etch-and-rinse ones [3]. However, it is precisely this combination that is crucial, if you want to make meaningful conclusions. For self-etching dentin adhesives, to our knowledge, the triangulated data are not available. Qualitative evaluations including the ISO 10993-5 [12] recommended grading according to so-called reactivity indices are also rarely available [36].

The study hypothesis of our screening study:

- Self-etch adhesives show no different cytotoxicity in relation to etch-and-rinse adhesives regarding the triangulated evaluation.
- Sequentially applied substances have different cytotoxic effects other than single applied substances regarding the triangulated evaluation.
- Self-etch one step and self-etch two steps adhesives show no different cytotoxicity regarding the triangulated evaluation.

## 2. Materials and Methods

### 2.1. Materials and Cells

Six different dentin adhesives were evaluated; an overview of these test materials is shown in Table 1. The pH values refer to the manufacturer’s instructions.

**Figure 1:**
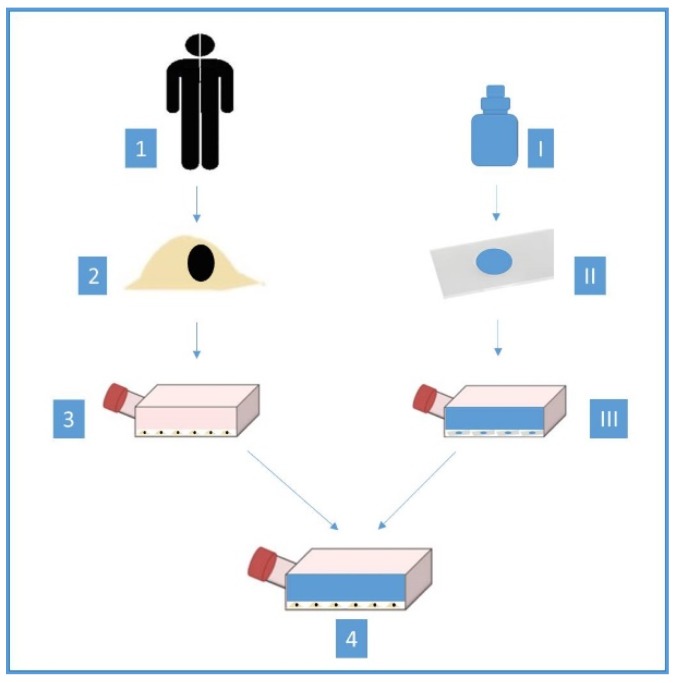
The method of the cytotoxicity assay (1 = biopsy of normal human gingival tissue, 2 = isolation of fibroblast cells, 3 = cultivation of fibroblast cells, I = different dentin adhesives, II = adhesives were applied centrally to the sterile bases of glass slides, III = eluate was obtained, 4 = eluate added to fibroblasts in Petri dishes).

Explants of normal human gingival tissue (Ethical approval code: 275/07) were obtained by surgical periodontal operation. Afterwards, this obtained basic material was stored overnight in the Hanks balanced salt solution (Gibco-Life Technologies Ltd., Paisley, Scotland) in a refrigerator at 4 °C, to cleanse the explants of blood and granulation tissue. They were supplemented with five millilitres bicarbonate and an antibiotic additive and cooled to 4 °C (Gibco-Life Technologies Ltd., Paisley, Scotland), to get germ poor conditions as possible. To prepare the human tissue for the test, they were cut with a sharp scalpel (No. 15, Aesculap, Tuttlingen, Germany) in uniform small pieces of 1 mm^3^. After that, the explants were transferred to surface-treated 50-cm^3^ polystyrene culture bottles and 50-mm-diameter polystyrene Petri dishes (Falcon, Becton and Dickinson, Heidelberg, Germany). The test tissue was dried for one to two minutes at room temperature. Afterwards, five millilitres of the culture medium (BM Eagle–Basal Medium) and 10% calf serum (both materials: Gibco, Paisley, Scotland, UK) were added to each Petri dish. Because oral human tissue cannot be obtained under sterile conditions, the germ content has to be reduced as much as possible with antibiotics. Penicillin was applied to every culture medium. The Petri dishes were incubated in a gas incubator (Nr, Heraeus, Hanau, Germany) at 37 °C with a 4.5% CO_2_ atmosphere and high humidity (95%). The culture medium was initially renewed every five to seven days and then after two to three days. The first proliferation of epithelial and fibroblast cells was present after 18 to 24 days around the explants and after two to three days a cell monolayer could be observed. For the present investigation, the test series was planned over a longer period and, therefore, a pure fibroblast culture was used, which was obtained by trypsinisation. The dentin adhesives were applied in the center to the sterile basses of glass slides under germ poor conditions. The glass slides with the single adhesive part remained uncured. Meanwhile, successively applied adhesive parts were cured together with the use of an Elipar II curing light (ESPE, Seefeld, Germany). The curing times are following the respective instruction for use (Table 1). These prepared glass slides were weighted and placed centrally into the Petri dishes. After that, the culture medium was applied into to Petri dishes to achieve a concentration of 0.2 milligram adhesive per one-milliliter medium. The extraction concentration has already used in earlier research and did not follow the ISO 10993-5 [3]. An eluate was obtained after 24 h. Five milliliters of this eluate were applied to fibroblasts in Petri dishes, which were 24 h old. They were between the 8th and 18th passage. Then, these Petri dishes were incubated at 37 °C in a 4.5% CO_2_ atmosphere for 24 h. Finally, the cell cultures were fixed with 98% pure ethanol and stained with Pappenheim’s panoptic stain. This method has already been used in earlier research [3,10] and is shown in Figure 1.

A total of 525 culture dishes were prepared, which resulted in 75 dishes per dentin adhesive respectively cell control. All cell cultures within a test series that were created under the influence of the dentin adhesives were compared with the cell control.

### 2.2. The Triangulated Parameters Tested

#### 2.2.1. Quantitative Evaluation

A total of 105 Petri dishes respectively 15 ones per group were used for the quantitative assessment, which were evaluated for “viable”, “dead” and “debris” after 24 h with a cell-counter (Cell-Counter CASY DT, OLS GmbH and Co KG, Bremen, Germany). It was adjusted after cursor setting for fibroblasts: viable: 12.8–100 µm; dead: 7.6–12.8 µm; debris: 3.3–7.7 µm. These values for “viable”, “dead” and “debris” fibroblasts are based on manufacturer recommendations and other studies already conducted [37].

#### 2.2.2. Qualitative Evaluation

In addition, a total of 420 Petri dishes, which resulted in 60 ones per group, were used for the qualitative analysis. They were examined at 100–250-fold magnification under a contrasting phase microscope (Leica, Bensheim, Germany), to identify physiological and pathological cellular changes. The general morphology, reactions and growth of the fibroblasts of the cells was evaluated, as well as any vacuolization, detachment and cell lysis that may occur. To document these cellular changes, 420 photos of the cell cultures were taken. One observer performed the assessment of the fibroblasts.

#### 2.2.3. Reactivity Index Evaluation

The reactivity index (Table 2) was determined based on qualitative assessment.

### 2.3. Statistical Analysis

The null hypothesis was tested by means of the Kruskal–Wallis multiple Conover–Iman–Tests and Bonferroni–Holm with adjusted significance level of alpha = 0.05 (BiAS.11.10, Epsilon, Frankfurt, Germany).

## 3. Results

### 3.1. Quantitative Result

Significantly less viable cells could be observed for the test materials One-up Bond F Plus (*p* = 0.0004), AdheSE (*p* = 0.01), Clearfil SE Bond (*p* = 0.002) in relation to the cell control. Comparing the material groups, hybrid bond (*p* = 0.04) and Syntac (*p* = 0.04) showed significantly more viable cells as One-up Bond F Plus. According to the dead and debris cells, no statistically significant differences could be found. The values for viable cells with respect to each dentin adhesive and the cell control have been summarised in Table 3.

**Figure 2:**
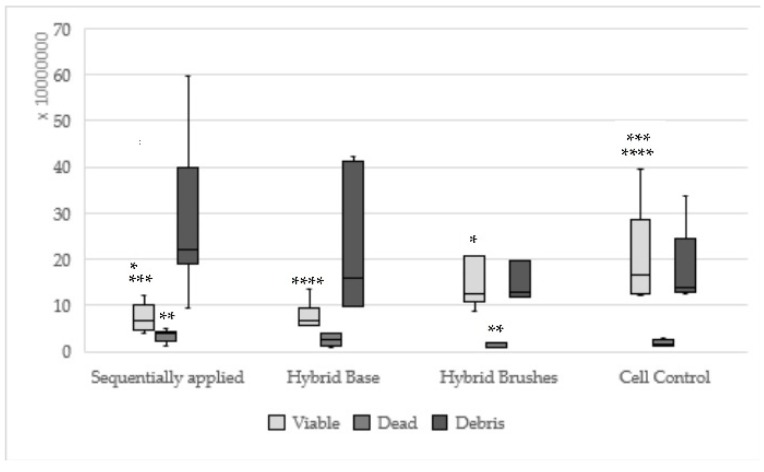
Results of hybrid bond sequentially applied, single applied (Hybrid base, hybrid brushes) and cell control; (sequentially applied vs. hybrid brushes * viable: *p* = 0.008 ** dead: *p* = 0.003; same asterisks show significant differences in the corresponding groups).

**Figure 3:**
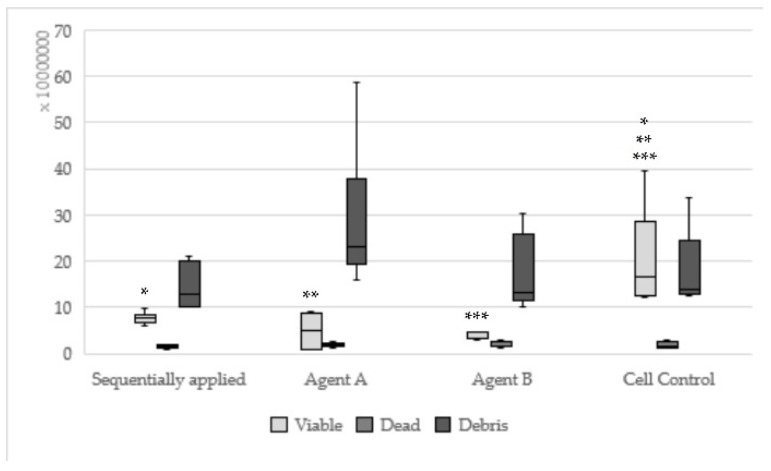
Results of One-up Bond F Plus sequentially applied, single applied (Agent A, Agent B) and Cell control (same asterisks show significant differnces in the corresponding groups).

**Figure 4:**
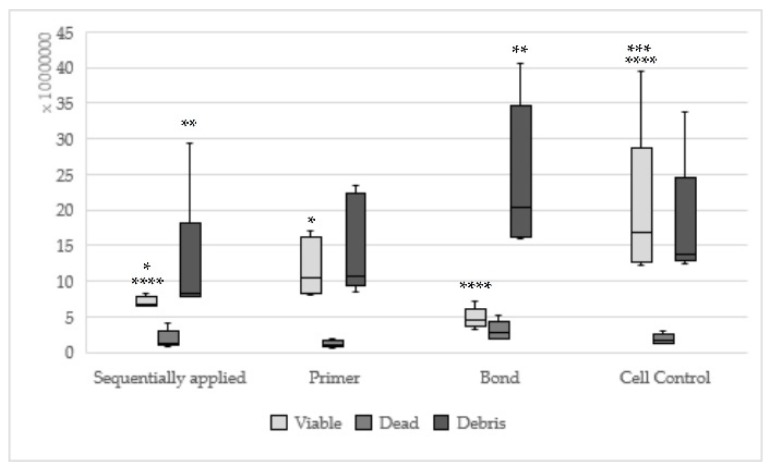
Results of AdheSE sequentially applied, single applied (Primer, Bond) and Cell control; (Sequentially applied vs. AdheSE Primer * viable: *p* = 0.008; sequentially applied vs. AdheSE Bond ** debris: *p* = 0.03; same asterisks show significant differnces in the corresponding groups).

**Figure 5:**
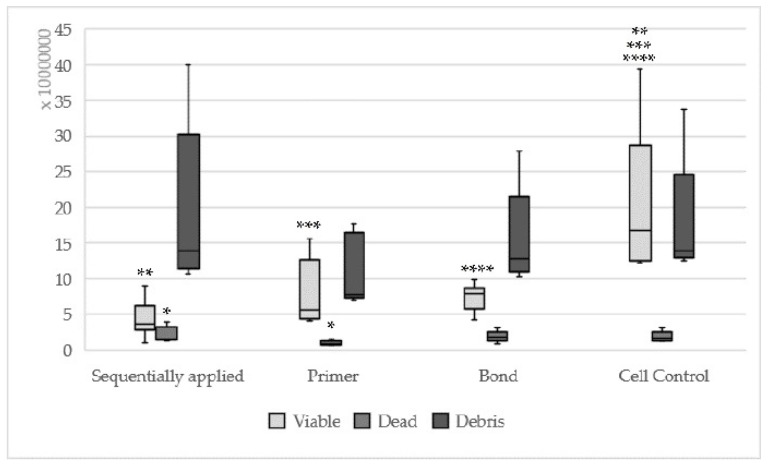
Results of Clearfil SE Bond sequentially applied, single applied (Primer, Bond) and Cell control; (Sequentially applied vs. Clearfil SE Bond Primer * dead: *p* = 0.04; same asterisks show significant differnces in the corresponding groups).

**Figure 6:**
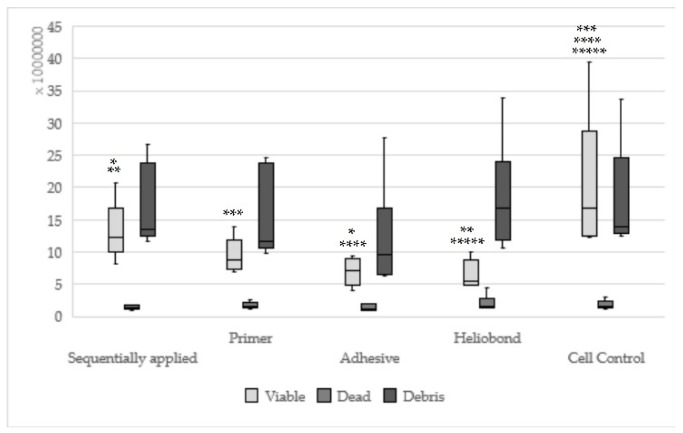
Results of Syntac sequentially applied, single applied (Primer, Adhesives, Heliobond) and Cell control; (Sequentially applied vs. Adhesive * viable: *p* = 0.005, sequentially applied vs. Heliobond ** viable: *p* = 0.002; same asterisks show significant differnces in the corresponding groups).

Hybrid bond sequentially applied as manufacturers recommend could be found statistically significantly less viable (*p* = 0.008) and more dead cells (*p* = 0.003) in relation to hybrid brushes solely (Figure 2). One-up Bond F Plus had no significant differences between sequentially and single applied adhesive parts (Figure 3). For AdheSE, significantly more viable cells were found for the primer (*p* = 0.008) respectively significantly more debris cells for the bond (*p* = 0.03) used in a single application in comparison to sequentially applied adhesive parts (Figure 4). Sequentially applied Clearfil SE Bond showed more dead cells than the Clearfil SE Bond Primer solely (*p* = 0.04) (Figure 5). The difference according to the viable cells between Syntac sequentially applied was significant to Syntac Adhesive (*p* = 0.005) and Syntac Heliobond (*p* = 0.002) each single applied (Figure 6).

### 3.2. Qualitative Result

**Figure 7:**
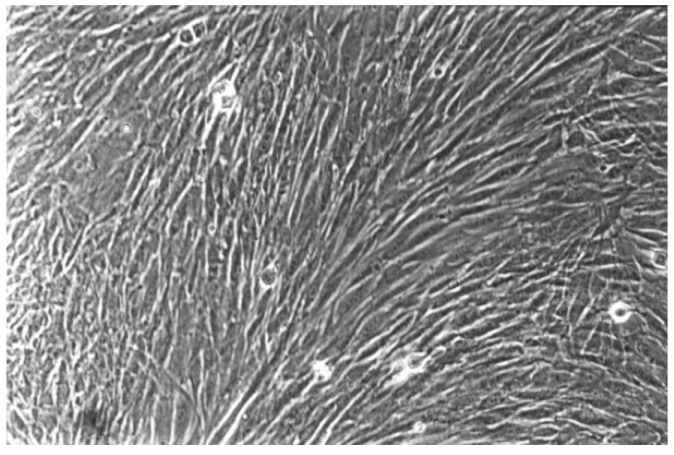
The cell control (No. 7) presents regular dense of fibroblasts with characteristic long cells and normal mitoses (100-fold magnification).

**Figure 8:**
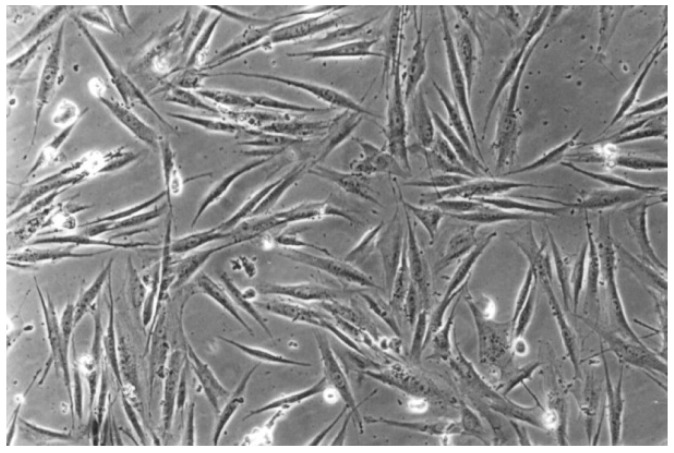
With Clearfil SE Bond (No. 4), the cell culture appears less dense than the cell control. Normal cells are predominating, but few rounded cells are found (100-fold magnification).

**Figure 9:**
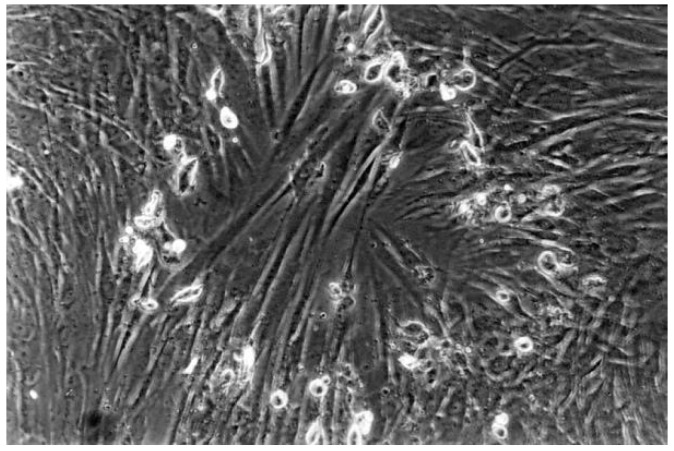
The contact cells to the material One-up Bond F Plus (No. 2) are rounded or dead. The fibroblast lawn is less dense than control cultures (100-fold magnification).

**Figure 10:**
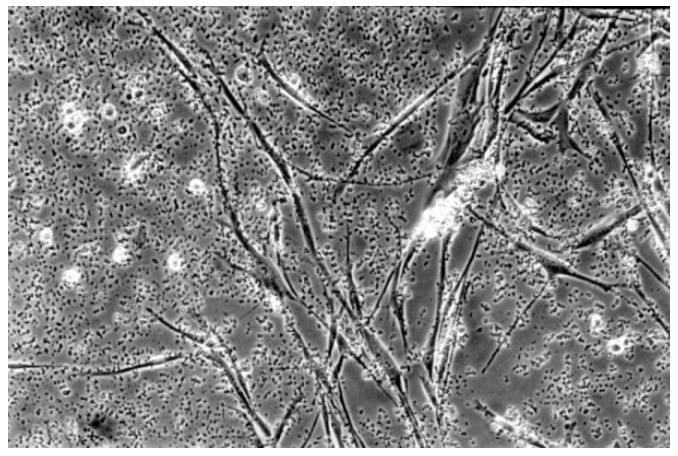
The cell culture exposed to dentin Adhesive Syntac (No. 5) is much less dense than the cell control, with many rounded and dead cells (100-fold magnification).

**Figure 11:**
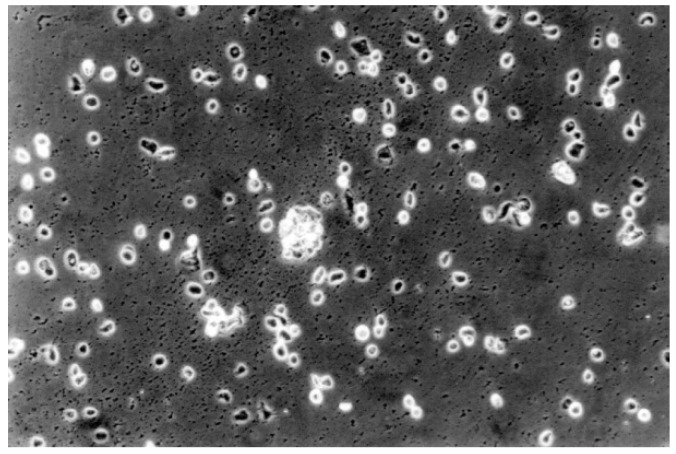
All fibroblasts under the influence of Optibond Solo Plus (No. 6) are in the process of dying. Many rounded and dead fibroblasts found already in the cell culture (100-fold magnification).

The qualitative evaluation is shown in Table 4, Table 5, Table 6, Table 7, Table 8 and Table 9. Figure 7, Figure 8, Figure 9, Figure 10 and Figure 11 showed the characteristic appearance of the cell cultures in the influence of the different dentin materials. The phenotype of this cell line has a characteristic morphology, which was observed with the use of light microscopy. This is characterized by spindle-shaped, long human primary fibroblast cells (pMF) derived from the gingiva human gingival primary fibroblast cells (HGPFC).

### 3.3. Reactivity Index Result

The reactivity index showed any statistically significant difference neither between Self-Etch and Etch-and-Rinse nor between the individual adhesives (Table 10). However, a statistically significant difference was found for this parameter between each dentin adhesive and the cell control.

Hybrid Bond sequentially applied showed a significantly higher reactivity index compared to Hybrid Brushes single applied (*p* < 0.1 × 10^−6^). Each single applied adhesive part One-up Bond F Plus Agent A (*p* = 0.1 × 10^−5^) and B (*p* = 0.02) had just significant differences compared to One-up Bond F Plus sequentially applied, which was evaluated with a lower reactivity index. It was found a significantly higher reactivity index for the AdheSE Bond single applied (*p* = 0.9 × 10^−4^) in comparison to AdheSE sequentially applied. Clearfil SE Bond sequentially applied showed a significantly higher reactivity index in comparison to both adhesive parts (Primer *p* = 0.0002; Bond *p* = 0.0009) single applied. A significantly higher reactivity index was found for Syntac sequentially applied in comparison to Syntac Primer (*p* = 0.002).

The null hypothesis H_0_^1^, in which self-etch adhesives showed no differences in relation to Etch-and-Rinse adhesives, can be accepted, in terms of the quantitative evaluation and the reactivity index. The null hypothesis H_0_^1^ is discarded regarding the quantitative results.

The null hypothesis H_0_^2^, that sequentially applied substances have different cytotoxic effects other than single applied substances, can be accepted. Except for One-up Bond F Plus, the null hypothesis is discarded regarding the quantitative results.

The null hypothesis H_0_^3^, that Self-Etch one step and Self-Etch two steps adhesives show no different cytotoxicity regarding the triangulated evaluation, can be accepted.

## 4. Discussion

Dental materials, which are applied for intraoral restorations come into contact with oral tissue and pulp cells, the cytotoxic effects on these cells are of high clinical importance [5,8,10,11,15,22,24,31]. Many different cell types were used to test the cytotoxicity of materials [10,11,14,15,16,17,18,19,20,21,22,23,24,25,26,27,28,29,30,31,32,33,34,35,36]. We decided to use primary human gingival fibroblasts, because they are closely related to the original tissue and thus are much better verifiable and more suitable to test cytotoxicity than cell lines [10,38,39]. Because the human tissue comes from just one donor, it is difficult to draw general conclusions. According to ISO 10993-5, a primary cell culture model can be used to test the cytotoxicity of dental material in vitro [12]. But it must be stated, that in vitro screening tests are limited to transfer to in vivo conditions, thus clinical studies cannot be replaced. In the oral cavity, the cytotoxic effects are influenced by saliva or microorganisms among other factors. These conditions cannot be created in an in vitro study. Additionally, it must be stated, that the extraction concentration of 0.2 milligram adhesive per one-millilitre medium has already used in earlier research and did not follow the ISO 10993-5, because none of the descriptions given in ISO 10993-12 corresponds to dentin adhesives [3].

One important finding of our screening study is that Self-Etch and Etch-and-Rinse adhesives showed no statistically significant difference in the observed cytotoxic effects in terms of the qualitative evaluation and the reactivity index. This is in accordance with other studies [14,15,16,17,18,19]. Self-etch dentin adhesives showed more cytotoxicity regarding the quantitative evaluation, which is also in line with previous studies [14,24,26,27,28]. Contradictory, other studies found that Etch-and-Rinse adhesives were more cytotoxic [11,17,20,21,22,23,24,25]. However, in all of the comparative studies, other methods, materials and their combinations were used [11,14,15,16,17,18,19,20,21,22,23,24,25,26,27,28]. It should be noted that in the clinical application of Etch-and-Rinse adhesives conditioning with 37% phosphoric acid is necessary. This step has not been tested, because there are already numerous studies regarding this topic [40,41,42]. The methods used to evaluate the cytotoxicity are categorized in the assessments of cell damage by morphological means, measurements of cell damage, of cell growth or of specific aspects of cellular metabolism according to ISO 10993-5 [12]. Most studies are based on settings, which are recommended by the ISO 10993-5 under further test procedures to evaluate the cellular metabolism such as MTT or XTT [14,15,16,18,20,21,23,24,28,29,30,31,32]. An MTT Assay is limited in the distinction of cells that are necrosis or altered into apoptosis [10]. In the present observational study, in which the morphological changes of the cells are evaluated, these differentiations are possible. MTT or XXT Assays require a previously performed screening according to the ISO 10993-5 [12]. Unfortunately, the number of screenings in this field is limited. Therefore, our aim was to perform our defined questions in the first step in a triangular setting with a screening study. According to ISO 10993-5, screening studies are performed when cells are examined under the microscope for changes in general morphology, vacuolization, detachment and cell lysis. These cell changes are represented by degrees in the reactivity index. As a quantitative assessment the measurement of cell numbers, such as dead cells, is recommended by an objective method [12]. In the present study, these guidelines were implemented by a qualitative evaluation with the help of a light microscope and the subsequent determination of the reactivity index and the quantitative assessment by means of cell-counters.

The cytotoxicity of the adhesives is influenced by the individual composition of ingredients. Significantly involved in this are the concentrations and combinations of the different monomers [43,44,45]. The cytotoxicity of monomers is ranked from the highest to the lowest: Bis-GMA, UDMA, TEGDMA, and HEMA [43,46,47]. This is not confirmed in our study regarding the quantitative evaluation with significantly differences. On the contrary, for One-up Bond F Plus significantly less viable cells were found compared to Syntac and Hybrid Bond. One-up Bond F Plus contains dimethacrylates in comparison to Syntac with a combination of TEGDMA, PEGDMA, Bis-GMA and HEMA. Only AdheSE, Clearfil SE Bond and One-up Bond F Plus showed a statistically significant difference to the cell control. They also contain dimethacrylates in their components, which has been the subject of debate in earlier research [24,34,48]. Dentin adhesive systems with higher cytotoxicity might contain more dimethacrylates of higher toxicity [34], but the dimethacrylates of the test materials are not described any closer from the manufacturers. Additionally, it must be stated that the dentin adhesives systems include other ingredients, e.g., different solvents, which contribute to their cytotoxicity [16,45]. In this study, the materials based on acetone, ethanol and water were tested. The water-based One-up Bond F Plus showed statistically significant less viable cells in relation to the acetone-based Syntac. Surprisingly, a statistically significant difference between One-up Bond F Plus and water-based Hybrid Bond was also stated. The other three water-based adhesives showed no significant differences to the above. As already explained, this might explain that the cytotoxicity is hardly influenced by the individual composition of the summation of their ingredients [16,45]. Contrary to the quantitative assessment, every adhesive showed a statistically significant difference to the cell control regarding the qualitative evaluation including the reactivity index. Thus, all the adhesives showed a cytotoxic effect, which is in accordance with previous studies [11,15,16,17,18,21,27,29,32]. The ranking of the cytotoxicity of monomers [43,46,47] cannot be confirmed with significant differences by the qualitative results and the reactivity index. It was reported, that Clearfil SE Bond showed a reactivity index of mild to moderate [16], which was also confirmed with our results. Syntac showed one of the highest reactivity indices compared to other test materials. In earlier studies, the cytotoxicity of Syntac was discussed controversial, because lower [33] and higher cytotoxicity [28,35] were found. It was already established in existing studies, that the Syntac containing glutaraldehyde has high cytotoxic potency [20,31]. Optibond Solo Plus showed the highest reactivity index, which could be induced by silicia in addition to the other ingredients [34]. The pH values of the dentin adhesives showed no significant differences between them, so that no conclusions can be drawn about their different cytotoxicity.

In terms of quantitative evaluation, all adhesives showed a statistically significant difference between sequentially and single applied except One-up Bond F Plus. Possibly, the number of individual components in a bottle might play a role, because the latter dentin adhesive contains only according to the manufacturer in Agent A four and Agent B three parts. Hybrid Brushes were statistically significantly less cytotoxic regarding viable and dead cells than the Hybrid Bond sequentially applied. The hybrid brushes contain no monomers, which might explain the low cytotoxicity. AdheSE Primer and Clearfil SE Bond Primer single applied were also significantly less cytotoxic than the adhesives sequentially applied. AdheSE showed this observation in terms of viable, Clearfil SE Bond of dead cells. Possibly, additional monomers or solvents respectively camphorquinone [34] could be a reason for this observation. Syntac sequentially applied was significantly less cytotoxic than Syntac Adhesive and Syntac Heliobond. It might play a role that these single applied adhesive parts have larger proportions of hydrophobic monomers like Bis-GMA [16] or also contain glutaraldehyde as the Syntac Primer, which also affected the cytotoxicity of Syntac sequentially applied [49]. Another reason might be the number of bottles, or indirectly the individual components of them, as already described. Because such an application comparison is published for the first time, the results cannot be compared with existing studies in the literature. There were also recognisable differences between the quantitative and quantitative results regarding the application comparison. One-up Bond F Plus showed a less reactivity index sequentially applied than the Agents A and B single applied. AdheSE sequentially applied showed a statistically significant lower reactivity index than AdheSE Bond single applied. It might be explained by the fact, that the sequentially applied adhesives were polymerized at the end as recommended by the manufacturers, whereas the single applied adhesive parts were uncured. In accordance with other studies, cured components show less cytotoxic effects than uncured ones [29].

The differences between quantitative and qualitative results respectively the reactivity index could be explained by the fact, that the cell-counter was calibrated with a specific setting for fibroblasts. The grading between “viable” and “dead” was classified only by cell size and conductivity [50]. The differentiation between the process of rounding off and the actual cell death is not possible. In contrast, the cell cultures were evaluated visually using microscopy, so that such differentiation can be evaluated in terms of qualitative results. Reactivity indices were determined on the basis of this. Since the experimental conditions of the quantitative method can best be transferred to further investigations, we consider this to be the most reliable method for evaluation.

In future research, the evaluation for these adhesives should be extended beyond 48 h and up to a long-term trial over 30 days, as described as longer extraction times in the ISO 10993-5 [12], like in other studies [15,21,24,27,35]. In order to further assimilate the cytotoxicity of the individual dentin adhesives, further investigation methods such as MTT or XTT Assay are planned after the screening. This investigation can also be supplemented by further screening studies with other adhesives to confirm the result of the comparison of the cytotoxicity of Self-Etch and Etch-and-Rinse adhesives.

## 5. Conclusions

In conclusion, the triangulated evaluation showed that the tested dentin adhesives were cytotoxic to the primary gingival fibroblasts. By limiting an in vitro study, only an adequate measure of the hazard potential can be provided. However, no differences in the cytotoxicity of the Self-Etch and Etch-and-Rinse adhesives could be demonstrated regarding the qualitative evaluation and the reactivity index, but the difference for the quantitative evaluation can be proved. There was also a difference in the cytotoxicity between sequentially applied and single applied adhesive parts.

## Figures and Tables

**Table 1:** Test material and their composition according to manufacturers.

No	Group	Dentin Adhesives	Manufactures	pH	Components	Curing Time
1	Self-etch, 1 step	Hybrid Bond	Sun medical, Moriyama, Japan	1.0	Part 1 Hybrid Base: Monomethacrylate, META, polyfunctional acrylate, water, acetone, photoinitiators, stabiliser Part 2 Hybrid Brushes: Sodium p-toluenesulfinate, aromatic amine	10 s
2	Self-etch, 1 step	One-up Bond F Plus	Tokuyama, Tokyo, Japan	1.17–1.26	Part 1 Bonding Agent A: Dimethacrylate, MMA, MAC-10, water Part 2 Bonding Agent B: DMAEMA, HEMA, MMA	10 s
3	Self-etch, 2 steps	AdheSE	Ivoclar Vivadent, Schaan, Liechtenstein	2.9	Part 1 Primer: Dimethacrylate, phosphoric acid acrylate, water, initiators, stabilisers Part 2 Bond: Dimethacrylate, HEMA, silicon dioxide, initiators, stabilisers	10 s
4	Self-etch, 2 steps	Clearfil SE Bond	Kuraray, Okayama, Japan	2.0	Part 1 Primer: HEMA, MDP, hydrophilic dimethacrylate, *N*,*N*-diethanol-p-toluidine, water, dl-camphorquinone Part 2 Bond: HEMA, MDP, Bis-GMA, hydrophobic dimethacrylate, *N*,*N*-diethanol-p-toluidine, silanated colloidal silica, dl-camphorquinone	10 s
5	Etch-and-rinse, 4 steps	Syntac	Ivoclar Vivadent, Schaan, Liechtenstein	2.5	Part 1 Primer: TEGDMA, PEGDMA, maleic acid, water, acetone, stabilisers Part 2 Adhesive: PEGDMA, glutaraldehyde, maleic acid, water Part 3 Heliobond: Bis-GMA, TEGDMA, stabilisers, catalysts	20 s
6	Etch-and-rinse, 2 steps	Optibond Solo Plus	Kerr, West Collins Orange, USA	2.4	Bis-GMA, HEMA, GPDM, water, ethanol, barium aluminoborosilicate glass, fumed silicia, sodium hexafluorosilicate, photoinitiator	20 s
7	Control: cell control			7.44		

Bis-GMA = bisphenol A-diglycidyl ether dimethacrylate, DMAEMA = dimethylaminoethyl methacrylate, GPDM= glycerol phosphate dimethacrylate, HEMA = 2-hydroxyethyl methacrylate, MAC-10 = 11-methacryloxy-1, 1-undecanedicarboxylic acid, MDP = 10-methacryloyloxydecyl dihydrogen phosphate, META= 4-methacryloyloxyetyl trimellitate anhydride, MMA = methyl methacrylate, PEGDMA = Polyethylene glycol dimethacrylate, TEGDMA = triethylene glycol dimethacrylate.

**Table 2:** Assessment of the reactivity index based on the ISO 10993-5 [12].

Grading	Reactivity	Condition of All Cultures
0	none	Discrete intracytoplasmatic granules, no cell lysis, no reduction of cell growth
1	slight	Not more than 20% of the cells are round, loosely attached and without intracytoplasmatic granules, or show changes in morphology; occasional lysedcells are present; only slight growth inhibition observable
2	mild	Not more than 50% of the cells are round, devoid of intracytoplasmatic granules, no extensive cell lysis; not more than 50% growth inhibition observable
3	moderate	Not more than 70% of the cell layers contain rounded cells or are lysed; cell layers not completely destroyed, but more than 50% growth inhibition observable
4	severe	Nearly complete or complete destruction of the cell layers

**Table 3:** Mean, Standard deviation (sd), minimum (min.), maximum (max.) and median values of the six dentin adhesives (viable cells).

No.	Dental Adhesive (1–6)	Mean	SD	Min.	Max.	Median	Significance in Rel. to No. *
1	Hybrid Bond	104,719,500	81,693,327	41,730,000	426,100,000	89,210,000	2
2	One-up Bond F Plus	55,844,444	27,706,721	9,969,000	96,830,000	64,050,000	1, 5, 7
3	AdheSE	76,550,000	37,983,471	29,010,000	170,300,000	72,060,000	7
4	Clearfil SE Bond	65,363,333	35,215,867	9,440,000	156,600,000	55,865,000	7
5	Syntac	90,745,000	38,932,657	39,580,000	206,700,000	83,495,000	2
6	Optibond Solo Plus	76,741,666	34,392,463	46,080,000	134,900,000	65,000,000	-
7	Cell Control	198,500,00	112,026,113	122,800,000	394,200,000	168,000,000	2, 3, 4

* The numbers indicate which pairs of groups showed a statistically significant difference (alpha = 0.05, Kruskal–Wallis multiple Conover–Iman–Tests and Bonferroni–Holm (BiAS.11.10, Epsilon, Frankfurt, Germany).

**Table 4:** Qualitative evaluation: hybrid bond (No. 1).

Concentration	Components Sequentially Applied	Components Single Applied	
		Hybrid Base	Hybrid Brushes
I	0.1–2.0 µL: the most fibroblasts rounded off, single vital fibroblasts	0.04–2.0 µL: almost all fibroblasts rounded off, few vital fibroblasts, 98%–100% cell death	0.1–1.0 mg: partly not so dense fibroblast grass, mitoses present, few retractions, similar to cell control
II	2.5–5.0 µL: 100% cell death	3.0–5.0 µL: 100% cell death	1.1–3.4 mg: similar to concentration I

**Table 5:** Qualitative evaluation: One-up Bond F Plus (No. 2).

Concentration	Components Sequentially Applied	Components Single Applied	
		Agent A	Agent B
I	1.0–6.0 µL: contact cells to the material all dead, many dead and rounded cells, fibroblast lawn less dense than cell control	1.0–4.0 µL: contact cells to the material all dead, remaining fibroblast lawn appearing normal	1.0–5.0 µL: material intensely distributed, fibroblast lawn less dense than cell control
II	7.0–14.0 µL: many rounded cells, few mitoses, fibroblast lawn much less than cell control	5.0–8.0 µL: fibroblast lawn less dense than cell control, rarely mitoses, cell death between 80 and 100%	6.0–10.0 µL: few small vital cells, many rounded cells, no mitoses, isolated vital fibroblasts, up to 100% cell death

**Table 6:** Qualitative evaluation: AdheSE (No. 3).

Concentration	Components Sequentially Applied	Components Single Applied	
		Primer	Bond
I	2.5–6.0 µL: vital fibroblasts, mitoses present, many rounded cells, fibroblast lawn less dense than cell control	5.0–9.0 µL: rounded cells, fibroblasts intensely vacuolated, mitoses present, fibroblast lawn less dense than cell control	3.0–4.0 µL: rounded cells present, few vital fibroblasts, dense fibroblast lawn on the Petri dishes margin
II	7.0–12.0 µL: dead cells, rounded fibroblast up to the petri dishes margin, material intensely distributed	10.0–14.0 µL: similar to concentration I	5.0–7.0 µL: fibroblasts vacuolated, dead cells, few small vital fibroblasts, no mitoses, cell dead between 75 and 100%

**Table 7:** Qualitative evaluation: Clearfil SE Bond (No. 4).

Concentration	Components Sequentially Applied	Components Single Applied	
		Primer	Bond
I	2.0–3.0 µL: small vital fibroblasts, no mitoses, dead cells, fibroblasts lawn less than cell control, material intensely distributed	4.0–5.0 µL: few small vital fibroblasts, no mitoses, rounded cells, dead cells present, material intensely distributed	3.0–4.0 µL: small vital fibroblasts, rounded cells, fibroblast lawn less dense than cell control
II	4.0–5.0 µL: the most cells rounded, few small vital fibroblasts, cell death between 80 and 100%	6.0–8.0 µL: rounded cells present, many dead fibroblasts, fibroblasts lawn much less than cell control, material intensely distributed	5.0–6.0 µL: small vital fibroblasts, fibroblasts vacuolated, rounded and dead cells present, fibroblasts lawn much less than cell control

**Table 8:** Qualitative evaluation: Syntac (No. 5).

Concentration	Components Sequentially Applied	Components Single Applied		
		Syntac Primer	Syntac Adhesive	Syntac Heliobond
I	0.1–1.0 µL: rounded cells present, vital fibroblasts, fibroblast lawn less dense than cell control	1.0–6.0 µL: rounded and dead fibroblasts, many normal fibroblasts	0.2–2.0 µL: whole petri dish with few small vital fibroblasts and rounded cells, between 95 and 100% cell death	1.0–5.0 µL: dead cells and rounded cells present, fibroblast lawn much less dense than cell control
II	2.0–2.5 µL: the most fibroblast rounded, 100% cell dead	6.0–12.0 µL: only in the transition zone fibroblast lawn much less dense, normal appeared cells, material zone sequentially grown	3.0–5.0 µL: 100% cell death	6.0–10.0 µL: at the Petri dishes margin normal vital fibroblasts, many rounded cells, fibroblast lawn much less dense than cell control

**Table 9:** Qualitative evaluation: Optibond Solo Plus (No. 6).

Concentration	Solo Plus
I	1.0–4.0 µL: small vital fibroblasts, many rounded cells, remaining fibroblast lawn less dense or as dense as the cell control
II	5.0–8.0 µL: rounded cells on the Petri dishes bottom, few vital fibroblasts at the Petri dishes margin, 95–100% cell dead

**Table 10:** Mean, standard deviation (sd), minimum (Min.), maximum (Max.) and median values of reactivity index.

No.	Dental Adhesive (1–6)	Mean	SD	Min.	Max.	Median	Significance in Rel. to No. *
1	Hybrid Bond	2.74	1.57	0.00	4.00	3.50	7
2	One-up Bond F Plus	2.98	0.86	1.00	4.00	3.00	7
3	AdheSE	2.96	0.87	1.00	4.00	3.00	7
4	Clearfil SE Bond	2.74	0.83	1.00	4.00	3.00	7
5	Syntac	3.02	0.83	1.00	4.00	3.00	7
6	Optibond Solo Plus	3.23	0.86	2.00	4.00	3.50	7
7	Cell Control	0.00	0.00	0.00	0.00	0.00	1, 2, 3, 4, 5, 6

* The numbers indicate which pairs of groups showed a statistically significant difference (alpha = 0.05, Kruskal-Wallis multiple Conover-Iman-Tests and Bonferroni- Holm (BiAS.11.10, Epsilon, Frankfurt, Germany).
